# Investigating Vascular Complexity and Neurogenic Alterations in Sectoral Regions of the Retina in Patients With Cognitive Impairment

**DOI:** 10.3389/fphys.2020.570412

**Published:** 2020-11-09

**Authors:** Delia Cabrera DeBuc, William J. Feuer, Patrice J. Persad, Gabor Mark Somfai, Maja Kostic, Susel Oropesa, Carlos Mendoza Santiesteban

**Affiliations:** ^1^Department of Ophthalmology, Bascom Palmer Eye Institute, University of Miami, Miami, FL, United States; ^2^Department of Ophthalmology, City Hospital Waid and Triemli, Zurich, Switzerland; ^3^Department of Ophthalmology, Semmelweis University, Budapest, Hungary

**Keywords:** retinal biomarkers, retinal vascular complexity, Alzheimer’s disease, cognitive impairment, fractal dimension, neurodegeneration, electroretinography

## Abstract

Evidence is accumulating that cognitive function, and visual impairment may be related. In this pilot study, we investigated whether multifractal dimension and lacunarity analyses performed in sectoral regions of the retina may reveal changes in patients with cognitive impairment (CI) that may be masked in the study considering the whole retinal branching pattern. Prospective age-matched subjects (*n* = 69) with and with no CI and without the presence of any ophthalmic history were recruited (age > 55+ years). The Montreal Cognitive Assessment (MoCA) was used to measure CI, and full-field electroretinogram (ERG) was performed. Also, visual performance exams were conducted using the Rabin cone contrast test (CCT). Quantification of the retinal structure was performed in retinal fundus images [45^*o*^ field of view (FOV), optic disk centered] with excellent quality for all individuals [19 healthy controls (HC) and 20 patients with CI] after evaluating the inclusion and exclusion criteria in all study participants recruited (*n* = 69). The skeletonized vasculature network that comprised the whole branching pattern observable in the full 45° FOV was obtained for each image and divided into nine equal regions (superotemporal, superior, superonasal, macular, optic disk, nasal, inferotemporal, inferior, and inferonasal). The multifractal behavior was analyzed by calculating the generalized dimension Dq (Do, D1, and D2), the lacunarity parameter (Λ), and singularity spectrum f(α) in the nine sectoral skeletonized images as well as in the skeletons that comprised the whole branching pattern observable in the full 45° FOV. The analyses were performed using the ImageJ program together with the FracLac plug-in. Independent sample *t*-tests or Mann Whitney *U* test and Pearson correlation coefficient were used to find associations between all parameters in both groups. The effect size (Cohen’s *d*) of the difference between both groups was also assessed. A *p*-value < 0.05 was considered statistically significant. Significant correlations between multifractal and Λ parameters with the MoCA and implicit time ERG-parameter were observed in the regional analysis. In contrast, no trend was found when considering the whole retinal branching pattern. Analysis of combined structural-functional parameters in sectoral regions of the retina, instead of individual retinal biomarkers, may provide a useful clinical marker of CI.

## Introduction

The impending growth in the number of people living with dementia will place significantly higher demands on both national and international healthcare (Hampel et al., 2018). Mainly, Alzheimer’s dementia is the most common form of dementia over the age of 65, affecting 5 million Americans (Hampel et al., 2018). There were 50 million people worldwide living with dementia with an estimated total cost of US$1 trillion in 2018, and researchers have estimated that there will be 82 million people worldwide living with dementia with an estimated total cost of US$2 trillion by 2030 (Hampel et al., 2018). The pathologic stamps of Alzheimer’s disease (AD) are beta-amyloid (Aß) plaques, neurofibrillary tangles (NFTs), and reactive gliosis (Koronyo-Hamaoui et al., 2011). As suggested by previous studies, AD begins decades before it is clinically expressed (Hampel et al., 2018). Therefore, a significant undertaking to prevent AD expression in individuals at high risk is identifying such persons who will eventually show the disease long before the early symptoms appear (Hampel et al., 2018). Current procedures such as magnetic resonance imaging (MRI), positron emission tomography (PET), and protein levels (e.g., tau and β amyloid) derived from cerebrospinal fluid (CSF) sampling tests are very costly and very invasive. They also lack specificity and are not readily accessible to most clinicians and affected people. The success of any future treatments for cognitive disorders will depend mainly on the ability to achieve an early and accurate *in vivo* diagnosis. Thus, there is a pressing need to identify early cognitive function changes that may precede symptom onset and predict eventual cognitive decline.

Previous studies have reported that CI complications are not limited to the brain but also include the retina. Such results are evident from the associations between the central nervous system (CNS) neurodegeneration and the loss of retinal ganglion cells and reduced bioelectrical activity of the retinal neurons (Katz and Rimmer, 1989; Krasodomska et al., 2010; Koronyo-Hamaoui et al., 2011; Snyder et al., 2016; Cabrera DeBuc et al., 2018, 2019; Arthur et al., 2019; Alber et al., 2020). Compared with standard neuroimaging techniques, a significant advantage is that retinal imaging is non-invasive and low priced. Consequently, because the retina reflects the CNS’s microenvironment, the human retina offers an alternate window to investigate and potentially identify the early development of CI.

Retinal vascular parameters such as fractal dimension (FD) reflect the optimality of the vascular network. As reported by several studies, retinal vascular network changes have been found in patients with CI (Cheung et al., 2014a,b; Alber et al., 2020). Particularly, a sparser retinal vascular network, represented by reduced arteriolar and venular FDs, has been associated with CI. Our previous pilot studies considered the whole retinal branching pattern to investigate the correlation between retinal vascular complexity and neurodegenerative changes in CI (Cabrera DeBuc et al., 2018; Arthur et al., 2019). The monofractal analysis is limited in describing the overall complexity of the vessel branching structure of the human retina. Therefore, multifractal analysis of the human retina has been employed because it can capture fractal properties at different scales providing more fine-grained insights into this structure as multifractals can provide information on the retinal geometrical features and their spatial distribution (Family et al., 1989; Mainster, 1990; Kyriacos et al., 1997; Stosic and Stosic, 2006; Ţălu, 2013a,b). These prior studies have demonstrated that the human retinal vessel structures are geometrical multifractals. Therefore, provided the multifractal metrics warrants describing the retinal vessel topology in a more in-depth manner compared to a monofractal, our studies relied on multifractal analyses. It is worth noticing that cognitive impairment (CI), as a clinical symptom, can be the result of many neurodegenerative diseases or age-related changes. Thus, these studies aimed to evaluate a low-cost and multimodal approach to investigate potential retinal biomarkers associated with CI complications, independently of mechanistic or causal relationships. The studies demonstrated that there are combined structural-functional metrics instead of individual biomarkers that may be sensitive or associated with CI. Specifically, we found a statistical trend pointing to the correlation between retinal vasculature changes and neuronal dysfunction suggesting that retinal geometric vascular and functional parameters might be associated with physiological changes in the retina of individuals with CI. However, these studies revealed a lack of significant “unique” associations between vascular and functional parameters while controlling for the other covariates, which suggested the need to conduct a regional analysis instead of considering the whole retinal branching pattern.

In this pilot study, we first investigated whether fractal complexity and lacunarity analyses performed in sectoral regions of the retina may reveal alterations in patients with CI that may be masked in the study when considering the whole retinal branching pattern. Second, we investigated how the vascular network complexity and neural function alterations in these sectorial regions of the retina may contribute to cognitive function differences. The central hypothesis is that multivariate retinal biomarkers in the sectorial areas reflect distinctive eye-brain signatures of CI that might have significant “unique” associations with CI’s onset and progression.

## Materials and Methods

Data collected in a previous study was reanalyzed in sectoral regions of the retina (Cabrera DeBuc et al., 2018). The study adhered to the Declaration of Helsinki, and it was approved by the Human Research Ethics Committee of the University of Miami. Informed consent was obtained from all participants following a thorough explanation of all test procedures. The approval was also achieved through a proxy for individuals who cannot understand information and make decisions due to their cognition status. Potential subjects with CI were identified from the clinic or recruited from a population attending adult care centers and community clinics using available medical records [e.g., Clinical Dementia Rating scale (CDR), mini-mental state examination (MMSE), and neuropsychological tests] (McKhann et al., 2011). The most recent results of the neuropsychological or screening tests could be over 1 year for some of the patients with mild or moderate CI. Therefore, we conducted the Montreal Cognitive Assessment (MoCA) test at the time of recruitment to (1) eliminate any potential time gap between cognitive and ocular assessments and (2) facilitate a standard screening metric for the cognition status that could be used in the analysis independently of the subjects’ CI causation or duration.

While complete details of the original methods (e.g., full details of inclusion/exclusion criteria, data quality control, statistical analysis design, confounding factors, ophthalmic assessments for ocular health, nomograms for retinal function, medication, and prior medical history) could be found in Cabrera DeBuc et al., 2018, herein, a very brief outline of the methodology is described to avoid rewriting the potentially duplicative content from our previous pilot study. The exclusion criteria were age under 55 years and the presence of any ophthalmic history before recruitment. Also, patients were excluded if they had other neurodegenerative pathologies. All study subjects underwent cognitive function assessment and electroretinogram (ERG) followed by advanced retinal imaging, color vision test, and visual performance exams of both eyes. All retinal regions were scrutinized for abnormalities, and subjects without any ocular history except for cataract surgery were included in the analyses. During the visual performance tests, all subjects wore their own best optical correction. Diabetes mellitus, hypertension, and cardiovascular disease were considered as comorbid medical conditions related to retinal vascular alterations. The history of study subject-reported smoking or current smoking was contemplated based on earlier reports that linked smoking with potential retinal vascular changes (Sun et al., 2009).

The MoCA was used to assess the short-term memory, visuospatial abilities, executive functions, language abilities, orientation to time and place, attention, concentration, and working memory in all study participants (Nasreddine et al., 2005). An experienced examiner administered the MoCA test in about 10–12 min. The MoCA total score range is 0–30, with lower scores (<26 points) indicating poorer cognitive ability. Patients with a score of ≥26 points are generally considered as having normal cognition with an average score of 27.4, compared with 22.1 in people with mild cognitive impairment (MCI) and 16.2 in people with AD (Folstein et al., 1975; Smith et al., 2007). The MoCA test also facilitates cognitive testing for visually impaired individuals. It also measures executive function, an essential dementia component not measured by the MMSE (Folstein et al., 1975).

Optic- disk centered images were acquired with a confocal scanning laser ophthalmoscope (cSLO, EasyScan, iOptics, Netherlands). The severity of color vision deficiency was tested using a tablet-based Cone Contrast Test unit (CCT, Provideo CCT Plus System, Innova Systems Inc., Burr Ridge, IL, United States; Rabin et al., 2011; Simunovic, 2016). The function of the retinal neurons was measured with a full-field (ERG, RETevalTM, LKC Technologies, Inc., Gaithersburg, MD, United States) according to the International Society for Clinical Electrophysiology of Vision (ISCEV) protocol (Marmor et al., 2004; Holder et al., 2007; Hood et al., 2008). The full-field ERG choice was mediated by the fact that this particular ERG protocol can be measured in a user-friendly manner using a low-cost and portable handheld device that does not require pupil dilation and facilitates measurements in the community or primary care settings with minimal training or no ophthalmic technician. It is also insensitive to moderate optical media opacities, a typical eye lens condition with aging. Also, prior work supports the full-field ERG’s clinical use in our study (Hinton et al., 1986; Porciatti and Falsini, 1993; Falsini et al., 1996; Devos et al., 2005). ERG amplitudes and implicit time values were measured consistent with the recommendations by the ISCEV (Mcculloch et al., 2015). The protocol used was the ISCEV 6 step, light-adapted first. Assessments consisted of light-adapted ERG (stimulus strength, 3.0 cd s/m^2^; frequency, 28.3 Hz flicker response). An extensive detailed methodology of the study could be found in Cabrera DeBuc et al. (2018), Arthur et al. (2019).

### Image Analysis Considering the Regional Retinal Branching Pattern vs. Whole Retinal Branching Pattern

**FIGURE 1:**
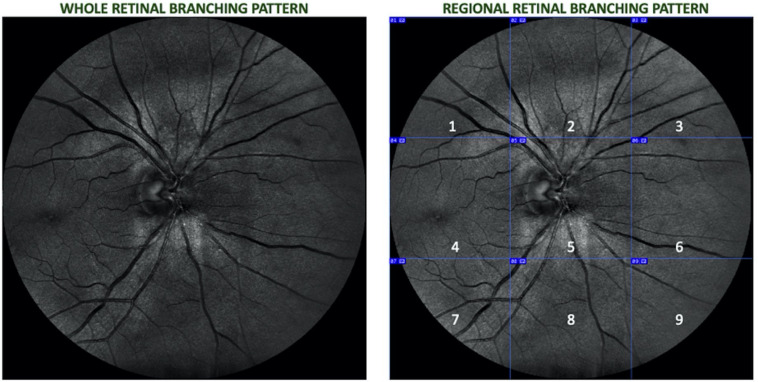
Retinal image of a right eye divided into nine equal regions (1 – superotemporal, 2 – superior, 3 – superonasal, 4 – macular, 5 – optic disk, 6 – nasal, 7 – inferotemporal, 8 – inferior, and 9 – inferonasal.

The optic- disk centered images were partitioned into nine equal regions observable in the full 45° field of view (FOV), and skeletonized using the public domain Java image-processing program ImageJ (Karperien, 1993). Figure 1 shows the vasculature network that comprised the whole branching pattern as it was first obtained for each image and later divided into nine equal regions (superotemporal, superior, superonasal, macular, optic disk, nasal, inferotemporal, inferior, and inferonasal). A more detailed explanation of the skeletonizing method and other related analyses can be found in Cabrera DeBuc et al., 2018.

### Lacunarity and Multifractal Analyses

The branching pattern of the retinal vascular network has been characterized by fractal and lacunarity analyses (Stosic and Stosic, 2006; Macgillivray et al., 2007; Ţălu, 2013a,b; Cheung et al., 2014a). The vascular FD is an average measure of complexity that describes “global” features of the whole branching pattern of the retinal vascular tree. Therefore, a more complex branching pattern denotes a larger FD value. The FD method has been widely used to characterize the retinal vascular network of patients with CI (Cheung et al., 2014a,b; Cabrera DeBuc et al., 2018). The multifractal behavior of the retinal vascular network is characterized by the generalized dimension spectrum (Dq vs. q, where Dq represents the FD at the *q*th order or exponent) and the singularity spectrum f(α) vs. α, which represents the whole spectrum of FDs (Bassingthwaighte et al., 1994; Stosic and Stosic, 2006; Costa and Nogueira, 2015; Ţălu et al., 2015; Zhu et al., 2019). Hence, *q* represents values from −10 to +10 with an increment of 1. These values are computed and then averaged with their standard deviations at each value of q. The Dq (e.g., D_0_, D_1,_ and D_2_) describes the multifractal characteristics of an object when condition D_0_ ≥ D_1_ ≥ D_2_ is satisfied, being D_0_, D_1,_ and D_2_ the capacity dimension, information or entropy dimension, and correlation dimension, respectively. The singularity spectrum for a multifractal object is typically a parabola with the concavity facing down and characterized by the height (Δf), width (Δα), and asymmetry (A; Shi et al., 2009). The more the Δα, the stronger is the multifractality, and the more complex is the pixel distribution within the image (Hu et al., 2009; Shi et al., 2009; Zhu et al., 2019). Also, greater values of the singularity exponents (α_0_, α_1_, and α_2_) of the singularity spectrum f(α) at *q* = 0, 1, 2 indicate greater singularities or maxima.

On the other hand, Λ is a parameter commonly used to describe the texture or coarseness of an image, and it can also differentiate two objects with similar FD (Faul et al., 2009; Ţălu et al., 2013; Zhu et al., 2019). Considering that the Λ parameter measures the heterogeneity or gap dispersion within an object, a high Λ describes an object with large gaps; while a low Λ is associated to homogeneous objects characterized by gaps that are all the same (Bassingthwaighte et al., 1994; Macgillivray et al., 2007; Faul et al., 2009; Zhu et al., 2019). The entire fractal analyses applied in this study using the segmented and skeletonized images of vessels have been previously described in detail in our previous studies (Cabrera DeBuc et al., 2018; Arthur et al., 2019). The Λ and fractal complexity analyses of the participants’ skeletonized images were conducted using the Image J software (Wayne Rasband, National Institutes of Health in Bethesda, Maryland, United States) together with the FracLac plugin with the specific settings as previously described (Karperien, 1993). A general detailed explanation, including mathematical formulas, of the lacunarity and fractal analyses, could be found in Cabrera DeBuc et al., 2018, and Arthur et al., 2019.

### Statistical Analysis

All statistical analyses were performed using IBM SPSS Statistics for Windows, Version 24.0 (IBM Corporation, Armonk, NY, United States). The Shapiro Wilk test of normality was used to test the Gaussian distribution of D_0_, D_1_, D_2_, α_0_, α_1_, α_2_, Δα, A, Δfα, and Λ Independent sample *t*-test was used to compare these parameters between the cognitively impaired and cognitively healthy participants assuming Levene’s test for equality of variance was not statistically significant. In cases where the Shapiro Wilk test was significant for any parameter, the Mann Whitney *U* test was used to compare that parameter between the two groups. Cohen’s d was used as an effect size measure when a parameter was significant when compared between the two groups [28]. A Cohen’s *d* value of 0.2 was considered small, 0.5 was considered medium, and ≥0.8 was considered large (Cohen, 1992). Pearson product-moment correlation was used to assess the relationship between the retinal multifractal parameters (D_0_, D_1_, D_2_, α_0_, α_1_, and α_2_), Λ and functional parameters (ERG’s IT and amplitude, and MoCA scores) for the cognitively impaired participants with a value ≥ 0.7 considered as high or a strong correlation. Also, correlations with a value < 0.7 were classified into ranges of importance: not statistically significant; significant, but weak, | *r*| < 0.32 (*r*^2^ < 10%; *p* < 0.05); modest, | *r*| between 0.32 and 0.55 (*r*^2^ from 10% to 30%); and moderate, | *r*| > 0.55 (*r*^2^ > 30%). A *p*-value < 0.05 was considered statistically significant. Logistic regression was used to construct predictive models to discriminate between phenotypes obtained from individuals with and without CI. Independent variables were divided into sets: (1) D_0_, D_1_, and D_2_; (2) α_0_, α_1_, and α_2_; (3) D_0_, D_1_, D_2_, α_0_, α_1_, and α_2_; (4) D_0_, D_1_, D_2_, α_0_, α_1_, α_2_, and Λ, and (5) D_0_, D_1_, D_2_, α_0_, α_1_, α_2_, and Λ; Flicker IT. Then, these series of five hierarchical models were fitted with all independent variables forced in. For each hierarchical set model, the logistic regression predicted probabilities of CI, computed from the model fitted coefficients and the measurements made for each case, were used as the test variable to construct Receiver Operating Characteristic (ROC) curves (Hanley and McNeil, 1982), in which the actual impairment status served as the state variable. In a separate analysis, all independent variables were allowed inclusion in a parsimonious forward stepwise fashion, and a ROC curve was also constructed with its model-predicted probabilities. Efficacy of discrimination was summarized with the area under the ROC curve (AUROC) and Youden’s index.

## Results

Data were reanalyzed from 20 individuals with CI and 19 healthy age-matched controls with normal cognitive function. These individuals were identified for the final analyses after exclusion criteria were revised for the 69 initially recruited participants (46% with CI). Specifically, individuals excluded had eyes with poor image quality, AMD, glaucoma, and diabetic retinopathy. Six subjects were pseudophakic but without any ocular history except for cataract surgery. All participants with diabetes mellitus (*n* = 9) and hypertension (*n* = 10) were well controlled and did not exhibit retinopathy signs. Also, the study subjects identified had mild and moderate CIs with a duration of 1–5 years after diagnosis, and the cause of their CI was unknown for some of the patients (Cabrera DeBuc et al., 2018). The study’s demographic characteristics were initially reported in Cabrera DeBuc et al. (2018). Cohen’s d was used as an effect size measure when a parameter was significant when compared between the two groups. By using Cohen’s d effect size, the regions and parameters with the greatest capacity to discriminate between control subjects and patients with CI were determined. But Cohen’s d was not computed in the absence of statistical significance (see Table 1). The correlation analyses were then conducted considering all variables altogether: functional (ERG parameters, MoCA scores) and statistically significant structural parameters obtained from the multifractal analysis, multifractal spectrum analysis, and lacunarity calculations (see Tables 1, 2). The functional ERG-IT photopic parameter was the parameter with statistical significance determined in the analysis conducted for the whole retinal branch pattern (Cabrera DeBuc et al., 2018), and the parameter used in the correlations in the regional analysis. In this study, a significant altered vascular network in individuals with CI (Tables 1, 2) was found when analyzing the data using both the regional and whole retinal branching pattern. Herein, a summary of the multifractal analysis results obtained in the previous studies is presented to facilitate the comparison with the regional analysis (Cabrera DeBuc et al., 2018; Arthur et al., 2019). In particular, smaller ERG’s amplitudes, larger ERG’s peak times, and drusen-like regions in the peripheral retina and pigment dispersion were noted in subjects with mild CI (Cabrera DeBuc et al., 2018). Likewise, the visual performance test with the computerized CCT revealed a functional loss in color vision, and that most individuals with CI had more green deficiency than red or blue deficiency (Cabrera DeBuc et al., 2018). Also, the participants with CI had lower singularity or α values than the cognitively healthy participants. Also, the Λ parameter was not significantly different between the participants with CI and the cognitively healthy subjects (Arthur et al., 2019).

**TABLE 1:** Multifractal dimension parameters and lacunarity values (mean + SD) of study participants with CI and cognitively healthy subjects for the analyses considering the regional and whole retinal branching pattern.

Multifractal dimension parameters	Cognitively healthy participants (*n* = 19)			Cognitively impaired participants (*n* = 20) (*p*-value, *Cohen’s d*)		
	Optic disk	Macular	Whole retina	Optic disk	Macular	Whole retina
D_0_	1.59 ± 0.05	1.50 ± 0.09	1.61 ± 0.03	1.52 ± 0.08 (0.006, *1.05*)	1.44 ± 0.16 (0.11, *NA*)	1.57 ± 0.06 (0.03, *0.84*)
D_1_	1.57 ± 0.05	1.48 ± 0.09	1.59 ± 0.03	1.50 ± 0.08 (0.005, *1.05*)	1.42 ± 0.15 (0.14, *NA*)	1.56 ± 0.06 (0.03, *0.63*)
D_2_	1.56 ± 0.05	1.46 ± 0.08	1.58 ± 0.03	1.49 ± 0.08 (0.004, *1.05*)	1.41 ± 0.15 (0.15, *NA*)	1.55 ± 0.06 (0.02, *0.63*)
Λ	0.31 ± 0.02	0.32 ± 0.05	0.34 ± 0.03	0.33 ± 0.04 (0.03, *0.63*)	0.32 ± 0.06 (0.73, *NA*)*	0.35 ± 0.05 (0.48, *NA*)

*Mann Whitney *U* test was performed; otherwise independent *t* test was performed. Generalized dimensions (D_0_, D_1_, D_2_) and lacunarity (Λ), NA: not applicable. Cohen’s *d* (values in *italics*) was not computed in the absence of statistical significance (reflected as *NA* in italics).

**TABLE 2:** Singularity spectrum parameters (mean ± SD) of study participants with CI and cognitively healthy subjects for the singularity spectrum analysis considering the regional and whole retinal branching pattern.

Singularity spectrum parameters	Cognitively healthy participants (*n* = 19)			Cognitively impaired participants (*n* = 20) (*p*-value, *Cohen’s d*)		
	Optic disk	Macular	Whole retina	Optic disk	Macular	Whole retina
α_0_	1.63 ± 0.05	1.55 ± 0.09	1.63 ± 0.03	1.57 ± 0.08 (0.01, *0.90*)	1.47 ± 0.17 (0.18, NA)*	1.60 ± 0.06 (0.03, *0.63*)
α_1_	1.57 ± 0.05	1.48 ± 0.09	1.59 ± 0.03	1.50 ± 0.08 (0.005, *1.05*)	1.42 ± 0.15 (0.14, *NA*)	1.56 ± 0.06 (0.03, *0.63*)
α_2_	1.55 ± 0.05	1.45 ± 0.08	1.58 ± 0.03	1.48 ± 0.08 (0.004, *1.05*)	1.40 ± 0.14 (0.15, *NA*)	1.54 ± 0.05 (0.02, *0.97*)
A	0.34 ± 0.07	0.42 ± 0.08	0.35 ± 0.05	0.37 ± 0.09 (0.36, *NA*)	0.38 ± 0.15 (0.29, *NA*)	0.35 ± 0.06 (0.99, *NA*)*
Δα	0.92 ± 0.10	0.94 ± 0.11	0.71 ± 0.05	0.95 ± 0.08 (0.51, *NA*)*	0.92 ± 0.14 (0.55, *NA*)	0.74 ± 0.05 (0.14, *NA*)
Δfα	1.05 ± 0.12	1.01 ± 0.13	0.87 ± 0.07	1.02 ± 0.12 (0.65, *NA*)*	1.06 ± 0.19 (0.37, *NA*)	0.87 ± 0.10 (0.93, *NA*)

*Mann Whitney *U* test was performed; otherwise independent *t* test was performed. Singularity exponents (α_0_, α_1_, and α_2_) of the *F* spectrum at *q* = 0, 1, 2, respectively. The Δf, Δα, and A represent the height, width, and asymmetry of the singularity spectrum, respectively. NA: not applicable. Cohen’s *d* (values in *italics*) was not computed in the absence of statistical significance (reflected as *NA* in italics).

The multifractal behavior satisfied the D_0_ > D_1_ > D_2_ criteria for both groups in the skeletonized images of the optic disk and macular regions and the whole retina, demonstrating the multifractal nature of the retinal vessel network. Statistical significance between retinal vascular and functional parameters was only observed for the optic disk and macula regions. Therefore, results are only reported for these two regions (Tables 1, 2). The D_0_, D_1_, and D_2_ values in the cognitively healthy group were significantly greater in the optic disk region vs. participants with CI (*p* < 0.05), respectively (Table 1). Table 2 shows similar trends in the optic disk region for α_0_, α_1_, and α_2_ in the singularity spectrum [f(α)] for the cognitively healthy group vs. participants with CI (*p* < 0.05). Similar trends were also obtained for the generalized fractal dimensions (Dq) and f(α) analyses in the whole retinal branching pattern. Still, the effect sizes (d) for the optic disk region were greater when compared with the whole retinal branching pattern (Tables 1, 2). The Λ values were greater for participants with CI than controls, *p* = 0.03 in the optic disk region, but this difference was absent in the whole retinal branching pattern and macular region, *p* = 0.48, and *p* = 0.73; respectively (Table 1).

The D_*o*,_ D_1,_ D_2_, and α_*o*,_ α_1,_ α_2_ values were significantly associated with MoCA scores and IT in the macular region (positive correlation), but these associations were absent in the optic disk region (Table 3). Also, the MoCA scores’ associations lacked in the whole retinal branching pattern (Table 3). Moreover, a significant negative correlation between the Λ parameter with the ERG-IT parameter was only observed when considering the whole retinal branching pattern (Table 4). Overall, the analyses revealed moderate (| *r*| > 0.55) and strong (| *r*| ≥ 0.7) correlation coefficients in the associations found for the D_*o*,_ D_1,_ D_2_, and α_*o*,_ α_1,_ α_2_ vs. IT parameters in the whole retina and macular region (Table 3). A modest correlation (| *r*| between 0.32 and 0.55) was obtained between the Λ parameter with the ERG-IT parameter in the whole retina (Table 3). In particular, the correlations obtained between the D_*o*,_ D_1,_ D_2_, and the ERG-IT parameter were higher in the macula region.

**TABLE 3:** Associations between retinal multifractal and functional parameters.

Parameters correlated	Optic disk region (Pearson coefficients, *p*-value)	Macular region (Pearson coefficients, *p*-value)	Whole retina (Pearson coefficients, *p*-value)
**Multifractal dimension parameters**			
D_0_ vs. MoCA	*r* = 0.22, *p* = 0.35	^†^*r* = 0.47, *p* = 0.04	*r* = 0.43, *p* = 0.06
D_1_ vs. MoCA	*r* = 0.21, *p* = 0.37	^†^*r* = 0.46, *p* = 0.04	*r* = 0.43, *p* = 0.06
D_2_ vs. MoCA	*r* = 0.21, *p* = 0.38	^†^*r* = 0.45, *p* = 0.04	*r* = 0.42, *p* = 0.06
D_0_ vs. IT	*r* = 0.40, *p* = 0.08	*r = 0.69, *p* = 0.001	*r = 0.64, *p* = 0.002
D_1_ vs. IT	*r* = 0.39, *p* = 0.09	*r = 0.69, *p* = 0.001	*r = 0.67, *p* = 0.001
D_2_ vs. IT	*r* = 0.40, *p* = 0.08	^‡^*r* = 0.70, *p* = 0.001	^∗^*r* = 0.69, *p* = 0.001
**f(α) Spectrum parameters**			
α_*o*_ vs. MoCA	*r* = 0.22, *p* = 0.34	^†^*r* = 0.46, *p* = 0.04	*r* = 0.43, *p* = 0.06
α_1_ vs. MoCA	*r* = 0.21, *p* = 0.37	^†^*r* = 0.46, *p* = 0.04	*r* = 0.43, *p* = 0.06
α_2_ vs. MoCA	*r* = 0.20, *p* = 0.39	^†^*r* = 0.44, *p* = 0.05	*r* = 0.42, *p* = 0.07
α_*o*_ vs. IT	*r* = 0.43, *p* = 0.06	*r = 0.69, *p* = 0.001	*r = 0.61, *p* = 0.004
α_1_ vs. IT	*r* = 0.39, *p* = 0.09	*r = 0.69, *p* = 0.001	*r = 0.67, *p* = 0.001
α_2_ vs. IT	*r* = 0.42, *p* = 0.07	*r = 0.69, *p* = 0.001	^‡^*r* = 0.71, *p* < 0.001
**Lacunarity parameters**			
Λ vs. MoCA	*r* = -0.25, *p* = 0.28	*r* = −0.24, *p* = 0.30	*r* = -0.18, *p* = 0.44
Λ vs. IT	*r* = -0.07, *p* = 0.77	*r* = -0.31, *p* = 0.19	^†^*r* = -0.51, *p* = 0.022

For abbreviations see Tables 1–2. P-values of less than 0.001 are represented as <0.001. ^†^Indicates a significant modest correlation (r from 0.32 to 0.55). ^‡^Indicates a significant strong correlation (|r| ≥ 0.7). *Indicates a significant moderate correlation (|r| > 0.55).

**TABLE 4:** ROC analysis results for CI detection using combined anatomical metrics from the optic disk region (D_*o*,_ D_1,_ D_2,_ α_*o*,_ α_1,_ α_2,_ and Λ) and functional measures (IT).

Model	AUROC	Std. error^*a*^	Asymptotic Sig.^*b*^	Asymptotic 95% confidence interval		Youden’s index
				Lower bound	Upper bound	
1. D_0_, D_1_, D_2_	0.734	0.080	0.012	0.577	0.891	0.44
2. α_0_, α_1_, α_2_	0.747	0.079	0.008	0.593	0.902	0.49
3. D_0_, D_1_, D_2,_ α_0_,α_1_, α_2_	0.879	0.060	0.000	0.762	0.996	0.75
4. D_0_, D_1_, D_2,_ α_0_, α_1_, α_2,_ Λ	0.871	0.062	0.000	0.749	0.993	0.75
5. D_0_, D_1_, D_2,_ α_0_, α_1_, α_2,_ Λ, IT	0.950	0.035	0.000	0.881	1.000	0.85

*^*a*^Under the nonparametric assumption.^*b*^Null hypothesis: true are = 0.5.*

**FIGURE 2:**
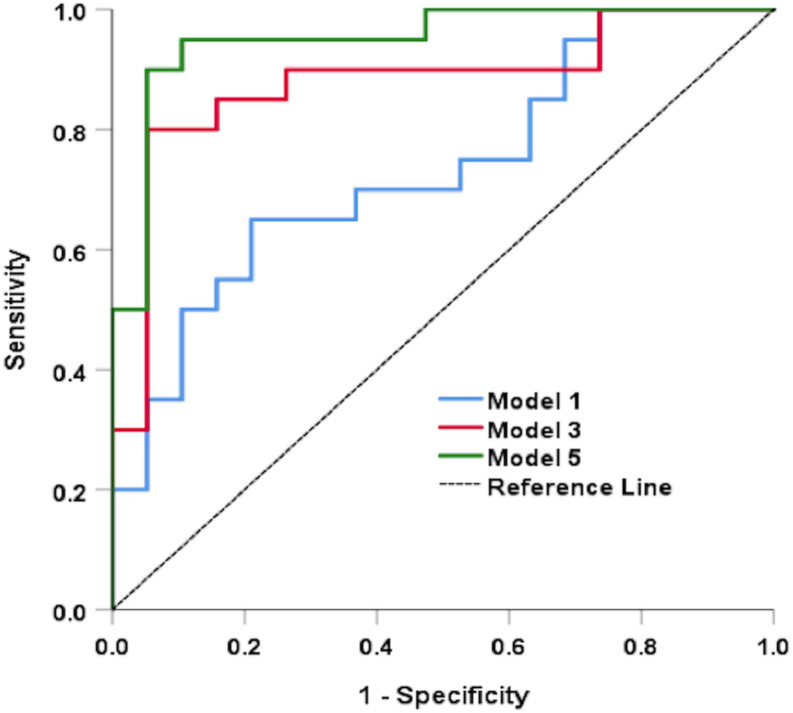
Results of the ROC analysis for CI detection using functional measures and multimodal anatomical metrics in the optic disk region. Models 1, 3, and 5 represents (D_0_, D_1_, and D_2_), (D_0_, D_1_, D_2,_ α_0_, α_1_, and α_2_), and (D_0_, D_1_, D_2,_ α_0_, α_1_, α_2_, Λ, and IT); respectively.

Figure 2 shows the ROC analysis results for CI detection using functional measures and multimodal anatomical metrics in the optic disk area, which was the sectoral region with a greater effect size (Cohen’s *d* value) when compared with the whole retinal branching pattern. According to the ROC analysis (see Table 4), the overall predictive accuracy of the multimodal (D_0_, D_1_, D_2,_ α_0_, α_1_, α_2_, and Λ; IT) predictive model in discriminating patients with CI from cognitively healthy subjects may be better (AUROC∼0.95) than that of the other combined measurements AUROC range ∼ [0.73–0.88]. In the separated analysis with all independent variables, the singularity exponent α_2_ was the most significant CI predictor. Once this was accounted for, none of the other parameters was statistically significant except IT. Therefore, α_2_ and IT were included in a single model to obtain a robust predictive index: [*Xlinear* = 18.387 + 0.736×*IT*−26.887×α2] being the Predictive probability of [CI=*e*^*Xlinear*^/1 + *e*^*Xlinear*^]. The AUROC for this predictive model was 0.90 (SE = 0.050) and was highly significant (*p* < 0.001).

## Discussion

This pilot study illustrates that there are potential multimodal retinal markers associated with CI complications. Our previous study couldn’t characterize the retina’s vasculature fractal properties fully (Cabrera DeBuc et al., 2018). Vascular patterns could be highly dissimilar and may have identical FD or be multifractal (Mandelbrot, 1977). Therefore, our study facilitated the regional characterization of retinal vasculature’s fractal properties entirely. Overall, our methodology takes advantage of the retinal vasculature network’s multifractal dimension, a metric that characterizes how optimal and efficient the retina’s blood distribution is (Rossitti and Löfgren, 1993; Murray, 2014). The retina multifractals are mixtures of monofractals that hold power-law relationships over several scales. Thus, we measured and detected such multifractals through a singularity spectrum, which provides valuable information on the retinal structure in a local region subset. We also took advantage of the Λ parameter, which can distinguish different fractal structures with the same FD. When combined and correlated with measured functional parameters (i.e., ERG and MoCA), these assessments could let us comprehend better the morphological and physiological changes that may result from symptoms such as CI.

Although vessel density analysis is widely used to characterize the retinal vasculature network, the density is not a scale-invariant metric (i.e., variations in vessel diameter can alter density). Therefore, a more efficient methodology was introduced to characterize the retinal vascular network by combining multifractal analysis with multifractal spectrum analysis and computing the Λ parameter. Our prior studies used the whole retinal branch pattern and revealed a lack of significant “unique” associations between vascular and functional parameters while controlling for the other covariates (Cabrera DeBuc et al., 2018; Arthur et al., 2019). However, our regional analysis uncovered significant differences in patients with CI that were masked in the study considering the whole retinal branching pattern. The Λ parameter was only significantly higher in the optic disk region in individuals with CI. However, compared to controls, the generalized dimensions and singularity spectrum parameters were significantly smaller in both the optic disk region and the whole retina in cognitively impaired subjects. These results demonstrated the reduced complexity of the retinal structure that might be associated with CI. Also, we found that there were significant differences in multifractal dimension and spectra, and lacunarity between the study groups in the optic disk region. Although these significant differences were observed cross-sectionally, showing smaller generalized FDs and larger Λ parameters, they may indicate a potential functional alteration from a space filling vasculature which nurtures the retina to a less dense vasculature as it degenerates in individuals with CI (Cheung et al., 2014a,b; Arthur et al., 2019; Alber et al., 2020). Our results also exposed that the sectoral region analysis revealed better the alterations in the multifractal analysis parameters for individual areas of the retina and their significant correlations with flicker-IT. Specifically, these alterations were characterized by a reduced complexity of the vascular network in subjects with CI. This trend indicates that the alteration is occurring from a space-filling vasculature network, which nurtures the retina to a less dense vasculature network as it degenerates in subjects with CI. This approach is expected to be especially useful for assessing subtle differences or abnormalities in the retinal function and vasculature network morphology of elderly subjects at risk of CI. Therefore, these results demonstrate the advantage of our quantitative approach compared to the prior studies for which only the generalized dimensions (D_*q*_) were used when considering the whole retinal branching pattern (Cabrera DeBuc et al., 2018).

As expected, our results showed that vascular patterns in a sectoral region with lower FD are characterized by higher Λ and shift of the singularity spectrum toward a lower α range and lower maxima compared to vascular patterns with smaller Λ. These results were in place for patients with CI suggesting a reduction of blood flow efficiency and impairment in circulatory transport due to a reduction from optimal vascular network architecture. The higher degree of “gappiness” observed in the retinal vasculature network of individuals with CI was only significantly different from the control individuals in the optic nerve region. This result may indicate decreased (collateral) circulation in this region with lower asymmetry caused by vessel remodeling showing a more available space (i.e., a higher gappiness) between the vessels. As reported in physiological coupling studies, and AD and other dementia-related studies, this particular gappiness might indicate a less effective neurovascular coupling (Berisha et al., 2007; Bashan et al., 2012; Querques et al., 2019; Mirzaei et al., 2020). The vascular structural alteration (i.e., gappiness) might be related to the reduced vascular demand in terms of nutrients and oxygen from the retinal neurons and the typical thinning of the peripapillary retinal nerve fiber layer (Hampel et al., 2018). Interestingly, this gappiness was also characterized by the large effect size found for the associations between the generalized dimensions and multifractal spectrum values with the ERG-IT in both the macular region and whole retina.

Our initial analyses suggested that our studies’ small sample size may have accounted for masked alterations in patients with CI when considering the whole retinal branching pattern (Cabrera DeBuc et al., 2018; Arthur et al., 2019). However, the sectoral region analyses show that the sample size did not affect the significant difference and large effect size found for the retinal vascular FD parameters, Λ, and multifractal spectra values between the two groups and the associations between the retinal vascular and functional parameters. However, the cause of CI for some of our patients is unknown – this could be due to the AD-high likelihood to unlikely due to AD (Albert et al., 2011). The causation inquiry requires a more comprehensive approach, including PET, MRI, and CSF analysis.

Although we have provided evidence of strong associations between fractal metrics and CI, it is compulsory to be conscious of our study’s limitations. First, our research has a cross-sectional design; therefore, designing longitudinal studies is essential for future analyses. Second, while the age and gender proportions were matched among the groups, our study group’s heterogeneity related to clinical characteristics demands to reproduce results in future research by including homogeneous groups of participants. We also note that unless patients who ultimately develop dementia are assessed before diagnosing the mild cognitive disorder, the actual duration of the MCI stage at the population level is not estimable. Thus, we couldn’t report information was not available at the time of recruitment and relied on the MoCA score obtained at the same time the ocular assessments were performed to facilitate a standard metric that was common for all study participants, including the healthy cognitively subjects. Larger sample size is also desirable to not doubt the interpretation of the results in future studies. Another shortcoming of our study is the potential bias due to our patients’ comorbidities, namely controlled hypertension and diabetes mellitus. Considering the age group of patients with CI, it is expected that roughly all subjects would have at least one of these conditions (Fryar et al., 2017; Centers for Disease Control and Prevention, 2020). Thus, it is challenging to isolate pure cases of CI. The retinal complications of hypertension, diabetes mellitus, and cardiovascular diseases have been described in detail earlier in the literature (Cheung et al.,2009,2012,2013; Grauslund et al., 2010; Sasongko et al., 2011; Sng et al., 2013; Ong et al., 2013; Broe et al., 2014; Popovic et al., 2018). However, there is much less information available on these pathologies’ effects on retinal vascular complexity, and even these data are somewhat contradictory. In the Singapore Malay Eye Study (SiMES), the vascular tree’s FD was not significantly different from that seen in subjects without diabetes; however, diabetic subjects had straighter arterioles (Cheung et al., 2012). On the contrary, the arterioles were more tortuous in subjects with diabetes than non-diabetic controls in another study (Sasongko et al., 2011). In a population-based young Danish cohort with type 1 diabetes, wider venular caliber, narrower arteriolar caliber, and lower FD measured at baseline were associated with the 16-year incidence of proliferative diabetic retinopathy, peripheral neuropathy, and nephropathy (Broe et al., 2014). In contrast, the FD was significantly associated with increased retinopathy odds in another study (Cheung et al., 2009). However, the macrovascular complications in another Danish study were not related to the FD of the retinal vascular tree (Grauslund et al., 2010). Of note, all these studies calculated the FD assuming the retinal vasculature as a monofractal structure. In a recent study, Popovic et al. (2018) described that the retinal vasculature’s FD was reduced in hypertensive retinopathy and proliferative diabetic retinopathy, using the STARE database. However, there was no difference observed between FD in hypertensive retinopathy and proliferative diabetic retinopathy (Popovic et al., 2018). Data from the Singapore Prospective Study Program indicated that a lower FD might be associated with higher mean arterial blood pressure and hypertension (Sng et al., 2013). Cardiovascular diseases can also influence the FD, as demonstrated by Cheung et al. (2013); Ong et al. (2013); both reporting a significant correlation between stroke and reduced FD. The above results obtained with the monofractal approach show that systemic diseases might influence the retinal FD; however, they are somewhat incongruent. Considering studies have used the multifractal approach like in our study, we found an OCTA study that reported no significant difference in the multifractal features measured in the superficial capillary plexus (SCP) between the mild NPDR and control groups (Ma et al., 2012). The SCP lacunarity was not found either significantly different between the groups. However, the deep capillary plexus (DCP) multifractal features and lacunarity were significantly different. But this study did not compare controls with diabetic subjects with no retinopathy because their main focus was on investigating relationships with the severity of diabetic retinopathy. In another study that used the same regional analysis with fundus images like in our study, the authors demonstrated that individual regions follow a multifractal behavior like in our study (Costa and Nogueira, 2015). Still, they couldn’t find significant differences in the retinal vascular network for either diabetic patients, with or without non-proliferative diabetic retinopathy when comparing the generalized dimensions, lacunarity, and singularity spectrum parameters. As Cheung et al. (2015) pointed, it is likely that microvascular complications in the eye influence retinal FD.

Overall, the study’s methodology could add additional investigative value to the use of retinal biomarkers to diagnose CI using a multimodal biomarker approach based on the retinal structure-function relationship. This approach has the advantage of a low-cost implementation in community settings. It could also be adapted to both detect the risk and early stage of AD and other neurodegenerative diseases with CI complications provided causation, and specific biomarkers are well-known in future studies.

## Conclusion

This pilot study introduced a more efficient methodology to characterize the retinal vascular network by combining multifractal analysis with multifractal spectrum analysis and computing the Λ parameter. The sectoral region analysis revealed better the alterations in the multifractal analysis parameters for individual retinal areas and their significant correlations with flicker-IT. Accordingly, instead of individual biomarkers, the investigation of combined multivariate vascular parameters in the retina’s sectoral regions might provide a useful clinical marker of CI. However, the full extent of our approach’s clinical applicability is provocative and still to be determined by using a larger and more diverse sample size with longitudinal study design. Overall, this pilot study would promote the investigation of multimodal, longitudinal models using the eye as a window to the brain to facilitate a low-cost approach for CI detection and change our understanding of CI.

## Data Availability Statement

The raw data supporting the conclusions of this article will be made available by the authors, without undue reservation.

## Ethics Statement

The studies involving human participants were reviewed and approved by Human Research Ethics Committee of the University of Miami. The patients/participants provided their written informed consent to participate in this study.

## Author Contributions

DCD conceived and designed the study. DCD, GS, MK, SO, and CMS performed the study. DCD, GS, SO, WF, and PP analyzed the data. DCD and GS contributed reagents, materials, and analysis tools. DCD and GS contributed to the writing of the manuscript. All authors contributed to the article and approved the submitted version.

## Conflict of Interest

The University of Miami and DCD have filed a Provisional patent application PCT/US19/64889. The remaining authors declare that the research was conducted in the absence of any commercial or financial relationships that could be construed as a potential conflict of interest.
